# Effects of Non-Polar Dietary and Endogenous Lipids on Gut Microbiota Alterations: The Role of Lipidomics

**DOI:** 10.3390/ijms23084070

**Published:** 2022-04-07

**Authors:** Konstantinos Tsiantas, Spyridon J. Konteles, Eftichia Kritsi, Vassilia J. Sinanoglou, Thalia Tsiaka, Panagiotis Zoumpoulakis

**Affiliations:** 1 Department of Food Science and Technology, University of West Attica, Ag. Spyridonos, 12243 Egaleo, Greece; ktsiantas@uniwa.gr (K.T.); skonteles@uniwa.gr (S.J.K.); ekritsi@uniwa.gr (E.K.); vsina@uniwa.gr (V.J.S.); 2 Institute of Chemical Biology, National Hellenic Research Foundation, 48, Vas. Constantinou Ave., 11635 Athens, Greece

**Keywords:** nutrition, gut microbiota, phytosterols, fat-soluble vitamins, carotenoids, eicosanoids, endocannabinoids, lipid mediators, lipidomics

## Abstract

Advances in sequencing technologies over the past 15 years have led to a substantially greater appreciation of the importance of the gut microbiome to the health of the host. Recent outcomes indicate that aspects of nutrition, especially lipids (exogenous or endogenous), can influence the gut microbiota composition and consequently, play an important role in the metabolic health of the host. Thus, there is an increasing interest in applying holistic analytical approaches, such as lipidomics, metabolomics, (meta)transcriptomics, (meta)genomics, and (meta)proteomics, to thoroughly study the gut microbiota and any possible interplay with nutritional or endogenous components. This review firstly summarizes the general background regarding the interactions between important non-polar dietary (i.e., sterols, fat-soluble vitamins, and carotenoids) or amphoteric endogenous (i.e., eicosanoids, endocannabinoids-eCBs, and specialized pro-resolving mediators-SPMs) lipids and gut microbiota. In the second stage, through the evaluation of a vast number of dietary clinical interventions, a comprehensive effort is made to highlight the role of the above lipid categories on gut microbiota and vice versa. In addition, the present status of lipidomics in current clinical interventions as well as their strengths and limitations are also presented. Indisputably, dietary lipids and most phytochemicals, such as sterols and carotenoids, can play an important role on the development of medical foods or nutraceuticals, as they exert prebiotic-like effects. On the other hand, endogenous lipids can be considered either prognostic indicators of symbiosis or dysbiosis or even play a role as specialized mediators through dietary interventions, which seem to be regulated by gut microbiota.

## 1. Introduction

Currently, more and more researchers are embracing the view that microbes are equally as important for the human body as cells. Among the systems that harbor microbes, the gut comprises the densest populated microenvironment, consisting of more than 3.8 × 10^13^ microorganisms [1], while the collected genetic material of all gut microorganisms constitutes the gut microbiome (GM). In addition, the human diet contains compounds (i.e., carotenoids, polyphenols, and dietary fibers), that are not digested by human enzymes, reaching the gut intact, where they are further catabolized by the microbiome, resulting in the production of unique metabolites. Interestingly, these gut-produced metabolites, along with the host’s other metabolites, shape the metabolic signature of the host, which can be mapped through the analyses of various biological fluids, such as urine, plasma, and feces. Taking into account the complexity of the GI tract, it is quite apparent that it is almost impossible to identify or quantify all the metabolites present in a biological sample. To date, state-of-the-art technological platforms (i.e., metabolomics, metagenomics, and transcriptomics) can be used in order to monitor and describe the unique and highly dynamic metabolic processes or pathways that occur in the human gut. Moreover, the implementation of -omics approaches enable the detection of a wide spectrum of different metabolites in various tissues [2,3]. 

One of the most important factors shaping the composition and, consequently, the properties of the intestinal microbiome is dietary lipids [4]. For instance, high-fat diets are suspected to play a role in the promotion of gut dysbiosis, which is defined as the imbalance of microbial populations in favor of pathogenic communities, while several dietary lipids (i.e., phytosterols and carotenoids) may reverse these effects [5,6]. Lipids are organic bio-molecules, which play a variety of important biological roles, such as energy saving, maintaining the integrity of membranes, and transporting and degrading other compounds. The term “lipidome” refers to (a) either the lipids originating from anabolic and catabolic pathways (endogenous lipids) or (b) the uptake of exogenous lipids through diet (dietary lipids), while “lipidomics” is a term used to present the current analytical framework applied in order to explain alterations that involve the lipidome [7]. 

Lipidomics provides new approaches to screen the metabolic pathways of lipids and therefore helps to understand lipid metabolism and its role in health and disease through the detection of lipid metabolites or other nutritional biomarkers [2,8]. In addition, considering the significant impact of diet in lipid metabolism, clinical lipidomics is a new integrative biomedicine field focused on the combination of lipid science with clinical medicine and nutrition [9]. This type of lipidomics is considered to be the answer to why certain types of diets, foods or even nutrients promote or inhibit the development of various gut-related diseases.

Application-wise, the combination of -omics techniques with high-throughput lipidomics can maximize their potential by developing tools which will help to achieve the desired comprehensive lipid analysis. However, it is essential to overcome specific limitations that may arise during experimental design or analysis. For example, the isomeric diversity of specific lipids (mostly fatty acids) as well as the differences between mass spectrometer ion sources need to be addressed in order to allow lipidomics to rapidly progress [10]. In addition, the lack of corresponding internal standards can be a real setback and may lead to quantitative inaccuracies due to the high sample complexity [11]. This is why an integrated, multifocal lipidomics platform must be very carefully designed in order to provide useful, reliable, and reproducible results and to extract as much information as possible. For that reason, targeted (determination of specific compounds) and untargeted (holistic) approaches, using GC/LC-MS^2^ techniques, are combined in metabolomics studies [10]. Regarding endogenous lipids, a new analytical field, known as lipid mediator (LM) metabolomics or metabololipidomics, is gaining ground. The expansion and implementation of this promising field will: (a) shed light on the pathways (biosynthesis or a biological role in inflammation) of bioactive lipids, suggesting novel pre-resolving mechanisms by which the host responds during inflammation, tissue damage, or the disturbance of homeostasis (gut dysbiosis) [12], (b) establish a benchmark for novel active resolution pharmacology approaches to control or even treat gut-related diseases, and (c) allow the direct correlation and assessment of the personalized metabolome with medicine and nutrition without the need for conjectures.

Despite the conflicting views that prevailed for years, lipids are now classified into eight major groups (1: fatty acyls-FA, 2: Glycerolipids-GL, 3: glycerophospholipids-GP, 4: sphingolipids-SP, 5: sterol lipids-ST, 6: prenol lipids-PR, 7: saccharolipids-SL, and 8: polyketides-PK) and several sub-classes (fatty acids, mono-, di-, or triglycerides, ceramides, isoprenoids, and acrylaminosugars). Assaying the impact of different dietary habits on configuring the intestinal microbial profile, the key role of lipid nutrients in host health management and in disease prevention must be underscored. Due to the Westernization of the human diet [13], researchers have scrutinized the effect of polar lipid intake, mainly fatty acids (i.e., ω-3, ω-6 PUFAs, MUFAs, etc.) and phospholipids, on the modification of gut microflora and on the maintenance of intestinal immunity and homeostasis [13]. The overall impact of an unhealthy nutritional lifestyle includes the increase in non-commensal (i.e., *Firmicutes* and *Proteobacteria*) bacteria, intestinal barrier dysfunctions, the decrease in gut microbiota diversity and intestinal immunity, the reduction in the mucus layer, the lower levels of bacteria-generated butyrate, and the stimulation of chronic inflammation pathways [14]. On the other hand, the balanced supplementation of phospholipids and the ω-3/ω-6 PUFAs ratio (in favor of the ω-3 fatty acids) increase the abundance of commensal bacteria (i.e., *Bifidobacterium*, *Akkermansia*) and reduce the *Firmicutes*-to*-Bacteroidetes* ratio (F/B ratio) [15], precluding the onset of various non-communicable gut-related disorders [16,17,18,19,20,21,22,23,24,25,26,27,28,29]. 

**Figure 1:**
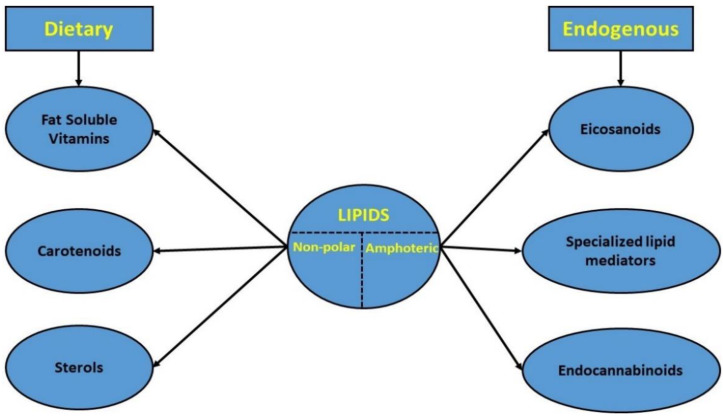
Classification of the studied lipid categories.

However, in this review, only non-polar dietary or amphoteric endogenous lipids were examined (Figure 1). This decision was based: (a) on the already existing huge amount of published data regarding gut-related interactions with more polar lipid categories, such as fatty acids, phospholipids, and short-chain fatty acids or cholesterol, and at the same time (b) on the lack of collective knowledge regarding the interrelationship of the lipids under study, gut microbiota, and host’s health state, which underlined the need for further investigation [13,15,30].

In particular, the dietary intake of these lipids could serve as a modulation strategy of gut microbiota functional ecology, to counteract any possible adverse health-related outcomes [30]. Nonetheless, data from both animal models and human interventions are still elusive and the effects of these nutrients are understudied. For example, despite the strong evidence that sterols (in particular phytosterols) affect the intestinal microbiome and the metabolism of the host by regulating microbiota composition (i.e., increase in *Bacteroides*, *Coprococcus*, *Oscillospira*, *Lactobacillus* and *Akkermansia* and decrease in *Desulfovibrio* genus, in a dose-dependent manner in the sterol-fed group), and cholesterol synthesis [31,32], the involved mechanisms and interactions have not been fully elucidated. Additionally, the metabolic fate and the effect on the intestinal microflora (and vice versa) of fat-soluble vitamins (FSVs) is still unclear. Recent findings show that this bidirectional relationship enhances important biological processes that take place in the gut (regulation, activation, and production of FSVs in the gut). In turn, these processes trigger many pivotal FSV-related functions, such as (i) the improvement of intestinal barrier integrity, (ii) the modulation of gut microbiota composition (i.e., increased *Proteobacteria* in the case of a high intake of vitamin D or increased *Sutterella* in the case of a lower intake of vitamin E), and (iii) the regulation of the immune and inflammatory response [33,34]. The landscape is similar for carotenoids. So far, carotenoids’ effect on gut microbiota composition has been investigated through (mainly) animal and human interventions focusing on specific metabolic diseases (i.e., obesity, diabetes type 2, etc.) or on diseases associated with metabolic syndromes, such as nonalcoholic fatty liver disease (NAFLD)). 

At the same time, even less is known regarding the interplay between amphoteric endogenous lipids (i.e., eicosanoids, endocannabinoids, and specialized pro-resolving lipid mediators (SPMs)), the gut microbiota, and nutrition patterns. At present, the research interest in such molecules is mainly focused on their ability to act as “mediators” during the manifestation of various inflammatory conditions, related to either the intestine or the various axes where gut microbiota participate (gut–brain, gut–retinal, gut–kidney, and gut–liver). In any case, nutrition remains the most important factor that regulates this bidirectional relationship. Therefore, the employment of high-throughput lipidomics is crucial in order to further investigate the role of endogenous lipids in the prο- and anti-inflammatory pathways, as well as to mark novel prognostic markers of gut function. 

Despite the increasing number of publications, the reciprocal relationship between lipids and gut microbiota must be further investigated in order to fill present knowledge gaps. Thus, the aim of the present comprehensive review is to unscramble the interrelation of nutrition and gut microbiome regulation, focusing on the role of non-polar dietary lipid nutrients and endogenous lipids by highlighting the use of lipidomic techniques. In detail, the sub-objectives of the current review are: (a) to review in depth the two-way interactions between dietary and endogenous lipids and the gut microbiota, (b) to evaluate the health impact of phytosterols, carotenoids, and lipophilic vitamins on the micromanagement of gut functional ecology, (c) to underline the use of lipidomics, implemented in several animal and human dietary clinical studies, for the elucidation of specialized biomarkers or endogenous mediators, and (d) to highlight the overall strengths and limitations of the up-to-date clinical studies. 

## 2. Review Methodology

**Figure 2:**
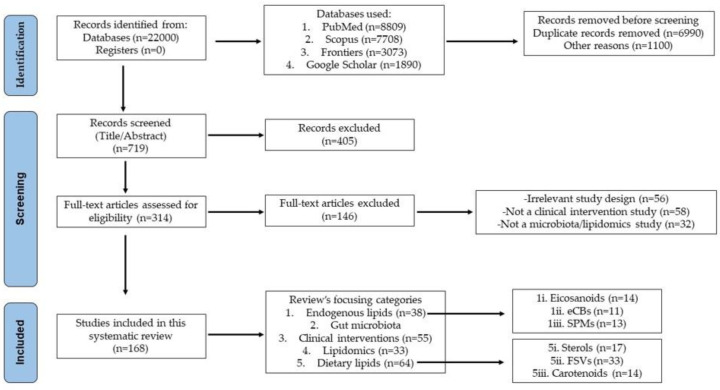
PRISMA flow diagram of the systematic review process.

Τhe adopted search strategy adopted and the method of article selection in this review were conducted in accordance with the Preferred Reporting Items for Systematic Reviews (PRISMA) statement (Figure 2). As a first step, the articles were evaluated on the basis of their title and abstract. The initial criteria for rejection or acceptance were defined as the presence or the absence of basic keywords (Figure 2) in the title. As a second step, the full text was also evaluated in terms of similarity to the main objectives of our study. With respect to studies published into 2021, the number of citations was also evaluated. Furthermore, in order to provide a comprehensive framework and remain impartial, four database sources, namely PubMed (Medline), Scopus (Elsevier), Google Scholar, and Frontiers (Health) were used. In addition, the search methodology was further divided into 5 categories according to the main objectives of the review (Figure 2), in which a combination of different keywords (i.e., gut microbiota, gut microbiota and dietary lipids, gut microbiota and (a) sterols, (b) phytosterols, (c) fat soluble vitamins, (d) carotenoids, (e) eicosanoids, (f) endocannabinoids, and (g) lipid mediators, and lipidomics and gut microbiota) and time frames was used, depending on the importance and timeliness of each. More specifically, for well-established scientific views or fundamental definitions, a ten-year time frame was chosen, while for specialized study subjects (i.e., clinical trials, interventions, and meta-analysis studies) a five-year time frame was selected.

## 3. Characteristics of the GI Microbiota

### 3.1. An Insight into Gut: What We Have Learnt So Far?

**Figure 3:**
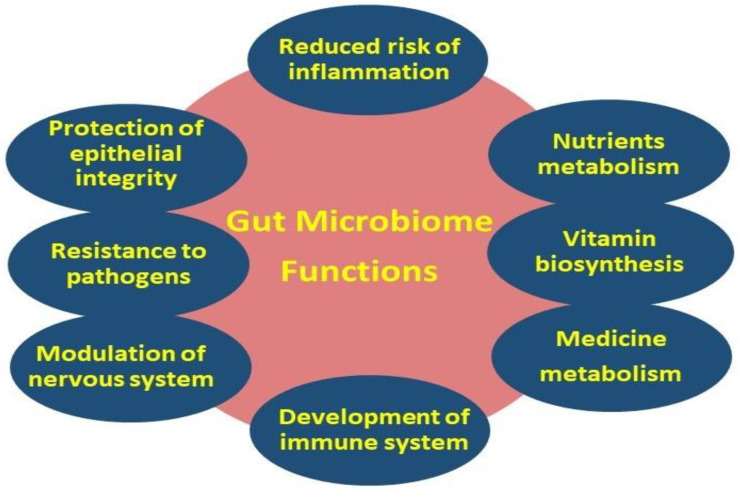
Primary (i.e., metabolism, gut–brain axis, and protection of epithelial integrity) and secondary (i.e., nutrients, vitamin and medicine metabolism, regulation of the immune and nervous systems, and resistance to pathogens) gut microbiome functions.

Although the definitions of the terms “microbiome” and “microbiota” are clearly different, these terms are commonly used interchangeably [35]. Nowadays, the study of the composition, structure, and functional properties of the human microbiome is a rapidly evolving scientific field. It is worth mentioning that the relationship between commensal bacterial and the host is an extremely dynamic system in which an intricate and mutually beneficial relationship, also known as symbiosis, is established [36]. The importance of this dynamic ecosystem is inextricably linked to various basic primary, as well as secondary functions, including the metabolism, immune system protection, the structural integrity of the epithelial barrier, and gut–brain axis communication [37] (Figure 3).

There is growing evidence that several gut disorders involve not only the GI system but distant organs as well [38]. Through a complex communication that includes the central nervous system as well as the autonomic and the intestinal nervous system, two-way interactions are created which affect both the gut microbiome and the lipids. Moreover, intestinal immune cells as well as the enteric nervous system affect the metabolism, absorption, and distribution of lipids, since they are key regulators of gut homeostasis [39]. Most recent studies link the gut with brain function (gut–brain axis), the host immune response, cell proliferation and vascularization, the regulation of intestinal endocrine functions, the modulation of energy biogenesis, the vitamin biosynthesis, and bile salts metabolism [40,41,42,43,44]. Focusing especially on lipid constituents, the gut–brain axis has the ability to regulate endogenous lipids (i.e., endocannabinoids, and SPMs) making them act “on demand” by exerting various bioactive properties, such as pro- or anti-inflammatory activities on the gut microbiota and immune system.

### 3.2. Gut Microbiota Stability and Composition: A Key Player in Various Gut-Related Diseases

As already stated, the gut microbiota presents a dynamic equilibrium that has adapted to harmoniously colonize the GI tract (symbiosis) [45]. Alteration in gut microbiota homeostasis can lead to undesirable situations, generally known as dysbiosis and abnormalities in the immune response of the intestinal microbiome. Gut dysbiosis is related to several chronic inflammatory conditions, also known as inflammatory bowel disease (IBD), including ulcerative colitis (UC) and Crohn’s Disease (CD). Moreover, various multi-factorial diseases or metabolic disorders (e.g., duodenum cancer, obesity, diabetes, and metabolic and immune-mediated disorders) are linked to microbial imbalances, which are also associated with the intake of lipids and their interactions with certain bacterial populations, highlighting the need to further investigate the underlying mechanisms [46].

Taking into account some unquestionable data regarding the structure, functionality, and anatomy of the GI system, it is widely accepted that the latter is divided into the stomach, small intestine, which is further divided into (a) duodenum, (b) jejunum, and (c) ileum, and large intestine (LI), which includes the colon and cecum. Every “compartment” is characterized by different conditions, such as pH, nutrient availability, or oxygen availability, and thus, each organ promotes the growth of specific microbes. Despite the fact that the gut environment favors the growth of bacteria from seven predominant phyla (e.g., *Firmicutes*, *Bacteroides*, *Actinobacteria*, *Fusobacteria*, *Proteobacteria*, *Verrucomicrobia*, and *Cyanobacteria*), its diversity is limited since more than 85% of the total population is constituted by *Bacteroides* and *Firmicutes* [47]. More specifically, the species of *Bacteroides* and *Firmicutes* phyla belong to the genera (a) *Bacteroides* and *Prevotella* and (b) *Clostridium*, *Eubacterium* and *Ruminococcus*, respectively. The major genus belonging to the phylum *Actinobacteria* in the human gut is *Bifidobacterium*, while *Actinobacteria* contribute to a small fraction of the total bacteria [48]. In Table 1, the different major phyla and bacterial genera that colonize each organ of the GI system are summarized. 

However, despite the various bacteria that colonize the GI system, even pathogen microorganisms can be found within it (i.e., *E. coli*, *H. pylori*, *C. jejuni*, *S. enterica*, and *B. fragili*) [54]. Furthermore, the fact that *Firmicutes* and *Bacteroides* are the predominant bacteria should not be considered as an infallible view, since significant differences can be observed in other phyla because of: (a) the current physio-pathological conditions, (b) the age (i.e., the microbiota is enriched during lactation and early years) and (c) the genetic background of the host, (d) the role of nutrition, and (e) geographical factors (i.e., levels of both *Firmicutes* and *Proteobacteria* were higher in European children, while *Firmicutes* were absent in West African children) [55].

## 4. Dietary (Exogenous) Non-Polar Lipids

As has already been stated, lipid supplementation through the diet can affect (a) gut microbiota composition, (b) the metabolic end products, (c) other enzymatic indicators (i.e., alkaline phosphate (ALP), aspartate transaminase (AST), alanine transaminase (ALT), and high- or low-density lipoproteins (HDL-LDL)), and thus (d) the fate of gut-related diseases [5,14,48]. In this direction, a thorough review of the literature was conducted in order to evaluate the relationship between the intestinal microbiome and dietary non-polar lipids, such as (phyto)sterols, fat-soluble vitamins, and carotenoids.

### 4.1. Dietary Sterols: Are They an Inducer of Gut Dysbiosis? 

Sterols, similarly to cholesterol, play an important role in the structure, integrity and properties of membranes. Phytosterols, which are plant-derived sterols, are found in abundance in Mediterranean diet models that promote beneficial changes in bacterial communities, while they are not present in a Western diet (high fat and cholesterol) [56]. In total, 20–80% of the cholesterol consumed daily (average recommended intake of 300 mg cholesterol per day) is absorbed, while the microbial absorption of phytosterols is only 2–3% (average intake of phytosterols is less than 500 mg per day) [57,58], which means that non-absorbed sterols can be further processed by the gut microbiome.

In particular, phytosterols are naturally occurring structural analogues of cholesterol, involved in altering certain lipid metabolic pathways. Thus, they are strongly related to the regulation of intestinal ecosystem and to the reduction in high hepatic cholesterol levels, which promotes gut dysbiosis in various liver abnormalities, such as steatosis, cirrhosis, liver failure, NASH, NAFLD, and hepatocellular carcinoma [59,60]. The manifestation of these pathologies is associated with the depletion of *Bacteroides* and *Bifidobacterium* and the increased richness of *Mucispirillum*, *Desulfovibrio*, *Anaerotruncus*, and *Desulfovibrionaceae*. Updated evidence has confirmed the detrimental effect of dietary cholesterol in microbial populations and in gut bacterial metabolites (taurocholic acid (TCA) and 3-indolepropionic acid (IPA)) [61]. 

Nonetheless, according to estimations, the dietary intake of PS (150–400 mg phytosterols/day) does not reach the necessary established levels (1500–3100 mg phytosterols/day) in order to exert its hypocholesterolemic effect (and receive the corresponding health claim). Consequently, the above-mentioned levels can only be achieved in the daily diet through PS-enriched foods, such as dairy products (PS-enriched milk, cheese, and fermented milk products). Focusing on in vitro studies, Cuevas-Tena et al. [62] investigated the impact of plant sterol enrichment dose on the gut microbiota of lean and obese subjects using an in vitro fermentation model, also known as TIM-2. In this study, the “PS-enriched” supplement, but also β-sitosterol alone, was able to increase the proportion of the genera belonging to the *Firmicutes* phylum. This increase suggested a potential modification of the short-chain fatty acids (SCFAs) and of the microbial profile of both lean and obese populations. However, the authors suggest that the daily intake of PS over several weeks and the different fecal inocula may lead to different effects on gut microbiota composition. One year earlier, the same research team revealed that the presence of PS during batch-culture fermentation led to a decrease in *Erysipelotrichaceae* species and an increment in *Eubacterium hallii* [63]. 

Meanwhile, another in vitro dynamic model was used in order to examine the impact of plant-sterol- and galactooligosaccharide-enriched beverages on colonic metabolism and composition [64]. According to the authors, a higher diversity in the gut microbiome was found in the transverse and descending colon, where the production of sterol metabolites (coprostanol, methylcoprostanol, and sitostenone) also took place. In addition, despite the fact that the prebiotic effect of galactooligosaccharides was not detected, alterations in gut microbiota (an increase in the *Parabacteroides* genus and the *Synergistaceae* and *Lachnospiraceae* families) denoted an enhancement of sterol metabolism. 

Furthermore, recent in vitro and in vivo studies confirmed that phytosterols, mainly β-sitosterol and stigmasterol, promoted gut symbiosis in cases of morbid obesity and hypercholesterolemia, by reducing the levels of the bacterial family *Erysipelotrichaceae* [65]. The supplementation of β-sitosterol in ruminants (sheep) lowered the abundance of the family *Lachnospiraceae* and increased the proportion of the genera *Prevotella* (*Bacteroidetes* phylum), presumably through the consequent increase in ruminal pH incited by the enrichment of the genus *Selenomonas* [66]. Although high-fat diets shift the F/B ratio toward the *Firmicutes* phylum in hamster models, plant sterols (i.e., soybean sterols) significantly attenuated this imbalance and improved gut microbiota diversity and richness of bacterial microenvironment (increase in *Bacteroides*, *Coprococcus*, *Oscillospira*, *Lactobacillus*, *Coprobacillus*, *Akkermansia*, and *Allobaculum* genera levels). The increased populations of these genera may present alleviating effects against high-fat-diet-related diseases, such as hypercholesterolemia and dyslipidemia [31,67].

Further intervention studies highlighted the potential modulating activity not only of free phytosterols, but also of their esters and their fully saturated derivatives, known as phytostanols. Namely, the relative abundance of *Anaerostipes* and *Bacteroidetes* species was increased in a high-dose diet of phytosterol esters (i.e., steryl esters). Phytosterol esters’ regulatory action was intertwined, via bile acid metabolism, with hepatic steatosis prevention in adult participants [68]. Sitostanol also increased the levels of *Bacteroidetes* communities, while campestanol uptake reduced the quantity of SCFA butyrate, produced by *Firmicutes* species in human clinical studies [5,68,69]. Apart from being dietary derivatives of phytosterols, 5α/β stanols (coprostanol, cholestenol, 5α/β-sitostanol, 5α/β-campestanol), detected in human feces, can also be gut-produced metabolites of sterols and, thus, potential biomarkers of bacterial metabolism [70]. 

In summary, although the exact associations of (phyto)sterols and the intestinal microbiome are still under study, there is enough evidence showing that these compounds are excellent regulators of cholesterol and potential modifiers of the gut microbiota composition. At the same time, even though the body of evidence regarding the impact of phytosterols on gut microbiota alterations and on diet-induced health or disease conditions is growing, there are a limited number of well-designed and controlled human studies. Since the current knowledge concerning the use of phytosterols as new therapeutic targets remains quite an unexplored domain, further focus is required to classify phytosterols as phyto-therapeutics in the foreseeable future [5,65].

### 4.2. Fat-Soluble Vitamins (FSVs): The Master Player in Nutrition–Gut Microbiome Tug-of-War

According to an increasingly large body of clinical findings, malnutrition, especially the low supply of non-energy-delivering micronutrients, such as vitamins, is negatively affecting the configuration of gut microbiota diversity and the intestinal health. Vitamin deficiency plays an important role in the pathogenesis of several diseases, namely neuropsychiatric disorders (depression, autism, Parkinson disease, schizophrenia, and multiple sclerosis), cardiometabolic disorders, complications of lipid metabolism (metabolic syndrome, obesity, and hepatic disease), and child development impairments in different age groups [14,71,72]. Of note, vitamins also manipulate the communities of the micro-ecosystems of mothers during pregnancy and of their offspring, both postpartum and during early childhood. For instance, vitamin D and retinol favor the growth of *Actinobacteria* and *Proteobacteria*, while vitamin E depleted them (mainly *Proteobacteria*) [73]. To date, mostly water-soluble vitamins (primarily those of B-group) have been in the spotlight of extensive research. However, many questions are left to be answered regarding the links between the intake or deficiency of fat-soluble vitamins, the resulting modification of the gut microbial ecosystem, and the contingent manifestation of various pathologies.

The Mediterranean diet is recommended as the ideal nutritional pattern in order to cope with the lack of FSVs, which are present in food items, such as vegetables, fruits, nuts, olive oil, dairy products, and fishes. The mutualistic interaction between vitamin uptake and gut microbiota composition is outlined with two different, yet firmly interrelated notions: (a) the impact of vitamins on shaping the microbial profile of pathogenic and nonpathogenic bacteria and (b) the role of microbiota in the synthesis, shuttling, and metabolism of vitamins and their metabolites [72]. Based on a brief overview of the impact of FSVs on microbial populations and health status control, the current data are quite controversial. On one hand, the administration of vitamins D, A, and K favored the prevalence of *Lactobacillus*. Nonetheless, in some cases, the intake of FSVs led to the increase of opportunistic pathogens or the depletion of synergistic bacteria belonging to several bacterial categories, such as *Proteobacteria*, *Deferribacteres*, *Enterobacteriacae*, *Clostridiaceae*, *Ruminococcus*, and *Odoribacter*, or *Verrucomicrobia*, *Bifidobacterium*, and symbiotic *Bacteroidetes*, respectively [14].

#### 4.2.1. Vitamin A

Vitamin A (retinol) and its enzymatic oxidation product (retinoic acid) play a key role in the intestinal immune response through interactions with the intestinal microbiome [74]. A sheep model confirmed the potential of vitamin A as a putative diagnostic indicator for male infertility. The abnormalities in its absorption were linked to the deregulation of bile acid metabolism, which is related to lower levels of *Ruminococcaceae* [75]. The inclusion of vitamin A in obesogenic diet patterns in three-week-old male C57BL/6J mice precluded changes in microbiota α-diversity and enriched the abundance of *Lachnospiraceae* [76]. Another study, targeting the investigation of gut microbiota alterations at different lifetime points, demonstrated that vitamin A insufficiency played a pivotal role in the embryonic but also in the early-stage development of four-week-old healthy rats. Especially in the periods of gestation, lactation, and weaning, the populations of *Diaphorobacter* and *Psychrobacter* (increase) or *Propionibacterium*, *Ochrobactrum*, *Enterobacter*, and *Staphylococcus* (increase) were affected. Τhe effect of vitamin A was imprinted in the serum metabolome by the presence of retinol, which presented a positive and a negative correlation with *Faecalibacterium* and *Staphylococcus*, respectively [77].

#### 4.2.2. Vitamin E

Vitamin E is considered a group of fat-soluble compounds and includes two main sub-categories: (a) α-, β-, γ-, and δ-tocopherols (TOHs) and (b) α-, β-, γ-, and δ-tocotrienols (T3), which are mainly presented in edible oils and several nuts [78]. Among these, a-tocopherol is one of the most important fat-soluble antioxidants of cellular membranes as it is the most biologically active form retrieved from human tissues. Additionally, it accounts for approximately 90% of the total vitamin E of the body [79].

In an experimental model, where five-week-old C57BL/6 male mice followed a high- and low-vitamin E diet, the phyla *Bacteroidetes* and *Verrucomicrobia* (*Akkermansia muciniphila* species) were related to lower body weight. More specifically, a dose-dependent relationship was highlighted between α-tocopherol and different gut microbial compositions, as the authors observed an increase in *Proteobacteria* and a decrease in *Verrucomicrobias* phylum [80]. Another study revealed that α-tocopherol supplementation was associated with changes in gut microbiota composition. Particularly, it was shown that a-tocopherol can reduce levels of *Bacteroides* and *Lactobacillaceae*, as well as the F/B ratio in humans [81]. δ-Tocotrienol, and its hydrogenated metabolite present in human feces, δTE-13′-carboxychromanol, can be considered as starting points against tumor growth [82]. Although they showed no significant effect on bacterial richness, they exhibited a modulating role in gut microbiota composition, by promoting the increase in health-promoting *Lactococcus* and *Bacteroides*. Focusing on δTE-13′-carboxychromanol, this tocotrienol metabolite counterbalanced the reduction in *Roseburia* in IBD patients and uniquely facilitated the elevation of *Eubacterium coprostanoloi* gene levels [82]. 

#### 4.2.3. Vitamin K

Vitamin K consists of vitamin K1 (phylloquinone, PKs) and vitamin K2 (menaquinone, MKs). Vitamin K1 is a naturally occurring compound in green leafy vegetables, as it is directly related to photosynthesis, while vitamin K2 is found in animal products. Apart from their intake through diet, menaquinones (ΜΚs) are also bacterial products of vitamin K, able to be remodeled in vivo. As proved by certain studies, vitamin K deficiency mostly affects female microbial composition with increased levels of *Lachnospiraceae* and *Ruminococcaceae* families [83]. A metagenomic analysis of the gut microbiota profiles of healthy volunteers and type 2 diabetes mellitus patients underlined the vital role of the phyla *Actinobacteria*, *Bacteroidetes*, and *Firmicutes*, mainly the *Erysipelotrichaceae* and *Corynebacterium* taxa, in the metabolic functionality of the diabetic gut microbiome related to the production of menaquinones [84]. According to the results of the aforementioned study, vitamin K2 emerged as a novel biomarker in the treatment of diabetes mellitus, also exerting other beneficial activities, such as enabling insoluble fiber digestion and refining immunomodulatory and nutritive molecules, such as SCFAs. Notably, MKs play a key role in gut microbiota homeostasis, promoting the growth of symbiotic bacteria. MK-7, one of the most studied vitamin K-related compounds, was reported to have protective effects against colon cancer during a study in male C57BL/6J mice [85]. In particular, the authors noticed a reduction in bacterial species promoting colorectal cancer, such as *Helicobacter apodemus*, *Helicobacter mesocricetorum*, *Allobaculum stercoricanis*, and *Adlercreutzia equolifaciens*.

#### 4.2.4. Vitamin D

Despite the well-known contribution of vitamin D to calcium homeostasis and bone health [86], the forms of this vitamin (calcitriol, cholecalciferol-vit-D3, and ergocalciferol-vit-D2) also participate in the regulation of: (a) blood pressure, (b) inflammation, (c) immune response, and, most recently, (d) gut microbiota [87,88,89,90]. Unlike vitamins A, E, and K, which were supplemented mainly in animal studies, vitamin D has a leading role, among lipid-soluble vitamins, in human clinical interventions. The aligned data in the literature provide a comprehensive insight into the crosstalk of the gut microbiota and vitamin D, primarily concerning the downregulation of inflammatory pathways. Though the effect of the gut microbiota signature on vitamin D metabolism is relatively established knowledge, the impact of vitamin D on gut microbial populations is still quite an uncharted field [91]. 

The administration of vitamin D in Crohn’s disease patients in remission positively affected bacterial taxa and the abundance of *Megasphaera* and *Lactobacillus*. However, no changes were observed in the gut microbiota diversity of ulcerative colitis (UC) patients, despite the major increase in *Enterobacteriaceae* [92]. Oral supplementation of vitamin D3 in a study including twenty adults resulted in a dose-dependent increase in serum D3 metabolite, 25-hydroxyvitamin D [25(OH)D]. Consequently, this led to the enrichment of *Bacteroides* and *Parabacteroides* abundance, which was associated with the alleviation of IBD symptoms [93]. However, seasonal sunshine variability (winter vs. summer) is responsible for the fluctuations in the levels of circulating 25-hydroxyvitamin D in IBD patients. Thus, a cohort study that evaluated the effect of seasons on the relationship between vitamin D levels and gut microbiota, covarying in intestinal metabolic derangements, suggested that higher levels of sunshine reduced pathogenic genera, such as *Fusobacterium*, *Collinsella aerofaciens*, *Eggerthella lenta*, *Bacteroides*, *Helicobacter*, *Faecalibacterium prausnitzii*, and *Rhodococcus*, *a*nd increased species of *Pediococcus*, *Clostridium*, and *Escherichia/Shigella* [94]. *Faecalibacterium* and *Akkermansia* species, which were increased after D3 intake, also influenced the immune responses and health status in autoimmune intestinal pathologies, such as UC syndromes [92].

As proved in in vivo studies (three-week-old male C57/bl6 mice) related to the microbiota–pain interrelationship, suboptimal levels of vitamin D resulted in a restricted microbial diversity and in an increase in F/B ratio [95]. A multi-vitamin dietary supplement, including vitamin D and vitamin B, was administrated in overweight individuals. Shifts were observed in one phylum (*Actinobacteria* decrease) and three families (*Actinomycetaceae*, *Bifidobacteriaceae*, and *Corynebacteriaceae* decrease) after vitamin D supplementation, and in three phyla (*Bacteroidetes* increase, *Cyanobacteria* and *Proteobacteria* decrease) and three families (*Christensenellaceae*, *Lachnospiraceae*, and *Enterobacteriaceae* decrease) after a combined vitamin D and B supplementation [96]. A cirrhotic rat model suggested that calcitriol, the active form of vitamin D3, controlled bacterial translocation and gut permeability and enriched the populations of *Bacteroidales*, *Allobaculum*, *Ruminococcaceae*, *Muribaculaceae*, and *Anaerovorax* [97]. Recent studies in NAFLD subjects verified the impact of vitamin D in the delay of cell death caused by inflammation, through the remodeling of the relative bacterial abundances in favor of *Lactobacillus* and against *Acetatifactor*, *Oscillibacter*, *and Flavonifractor* [98].

Based on official guidelines, vitamin D is an essential nutrient in pre- and post-natal maternal diet and infant formulas, as the infant microbiome is rapidly evolving and altering up till early childhood years. According to the results of the CHILD (Canadian Healthy Infant Longitudinal Development) cohort study, the supplementation of vitamin D to both formula-fed and exclusively or partially breastfed infants negatively affected the concentrations of the *Megamonas* genus. In the group of exclusive breastfeeding, a diet rich in vitamin D during pregnancy was related to higher populations of *Haemophilus* and lower populations of *Bilophila* and *Lachnospiraceae*, while no compositional changes in the gut microbiota of partially breastfed or formula-fed infants were observed. Even though vitamin D supplementation of the mother or infant was not directly linked to Clostridioides difficile colonization, the maternal intake of vitamin-D-fortified milk minimized the risk of *C. difficile* colonization in infants [99]. Aligned data from the current literature highlight the importance of the feeding regimen in the foundation and constitution of the gut ecosystem in infants. The additional supplementation of vitamin D in the breastfed group stimulated the farming of *Bifidobacterium*, which are known to act as probiotics. On the contrary, no significant differences were noted in the gut taxonomy of formula-fed infants with or without vitamin D supplementation [100]. 

Additionally, the lack of vitamin D, which induced the abundance of *Erysipelotrichaceae* and *Veillonellaceae*, is the most common marker in the cases of osteoporosis in postmenopausal women. Nonetheless, it was intriguing that the presence of vitamin D in serum disclosed a negative correlation with *Enterobacteriaceae* and *Erwinia*. In addition, higher concentrations of vitamin D were affiliated with the amino acid metabolism, particularly with higher levels of the metabolites alanine, proline, tyrosine, valine, and leucine [101]. While the focus of current dietary interventions concerns chronic disease cases, little is known about the gut-regulated individualized responsiveness of healthy female subjects to vitamin D intake. The fact that the deficiency of vitamin D can be responsible for fragile bone health is a common observation. According to studies related to the effect of vitamin D on women, the dominating commensal phylum *Bacteroidetes* and taxa *Akkermansia* and *Bifidobacterium* were more abundant after vitamin D supplementation. Moreover, the variations in the gut microbiota diversity of bacterial genera were more prominent in the group of individuals who responded to vitamin D supplements than in the non-responders group, where the concentrations of *Bacteroides acidifaciens* were decreased [102]. 

Furthermore, several studies pointed out that the administration of FSVs, in total, yielded beneficial outcomes with regard to the state of the health of neuropsychiatric patients, by orchestrating the balance between bad and good microbes, through their biosynthesis and their interaction with gut microbiota [71]. Based on the results of a pilot study in an older Australian population, all vitamins (hydrophilic and lipophilic) are colon-delivered micronutrients, which instigate modifications in (a) the phyla of *Actinobacteria* (increase with vitamin A) and *Bacteroidetes* (reduction with vitamin D3), (b) the families of *Coriobacteriaceae* (increase with vitamin A), *Ruminococcaceae*, *Peptostreptococcacea* (increase with vitamin D3), and *Desulfovibrionaceae* (slight decrease with vitamin D3), (c) the genera of *Collinsella*, species *aerofaciens* (slight increase with vitamin A and D3) and *Bilophila* (slight decrease with D3), and (d) the species *Collinsella aerofaciens* (slight increase with vitamin E) and *Eubacterium hallii*, *Coprococcus comes*, and *Dorea longicatena* (increase with vitamin D3) [103].

In light of the dietary interventions under review, FSVs are wielded in the manipulation and restoration of gut microbiota, compared with the other two non-polar nutrients included in the present review. Nonetheless, the elucidation of the reciprocal interactions between lipid-soluble micronutrients and the gut microenvironment merits further research, in order to entrench specific guidelines for FSV supplementation and implementation in novel therapeutic strategies.

### 4.3. Carotenoids: Can They Balance Diet–Gut Microbiota Crosstalk?

Carotenoids, an important subgroup of terpenoids, are minor dietary phytochemicals present in red fruits or vegetables (orange, peaches, tomatoes, carrots, pumpkins, and peppers) and in green leafy vegetables (broccoli, spinach, and kale). These natural pigments are divided into two major groups: (a) xanthophylls, such as lutein, zeaxanthin, astaxanthin, etc., which contain >1 oxygen atom, and (b) carotenes, such as α-carotene and β-carotene, which contain no oxygen atoms and are the major precursors of vitamin A. Clinical trials have shown that carotenoids in low levels demonstrate beneficial effects, while overdoses are toxic [104]. However, carotenoids can protect against age-related eye diseases, metabolic syndromes, cardiovascular diseases, diabetes, inflammation, and, most recently proven, body composition changes [105,106,107]. Interestingly, carotenoids cannot be synthesized by the human body and thus can only be obtained through the diet. As fat-soluble compounds, carotenoid bioavailability is considered low (10–40%). However, their lipophilicity renders their absorption by the GI tract more efficient compared with that of hydrophilic molecules. Despite the accumulated data dealing with the interplay of the gut microbiome and other lipid dietary constituents, the bidirectional relationship between gut microbiota populations and carotenoids is as of yet poorly understood. There is no solid evidence concerning the impact of microbiota (a) on carotenoids and their bacterial metabolites or (b) on the molecular triggers, which activate the beneficial health functions of carotenoids.

To date, a summarized effort in order to collect the most recent data regarding the interactions of carotenoids and the gut microbiota in both animal and human studies has been made (Table 2). It is worth mentioning that the majority of these studies focus on the impact that specific carotenoids have on certain intestinal-related diseases (e.g., obesity, NAFLD, and cancer-related diseases). Overall, carotenoids promote the increase in nonpathogenic bacteria, such as *Bifidobacterium* and *Lactobacillus*, and restore the balance of *Firmicutes/Bacteroides* fractions. More specifically, Xia et al. [108] showed that dietary tomato feeding, high in lycopene, prevents both high-fat diet (HFD)- or diethyl-nitrosamine (DEN)-induced inflammation through the potential modulation of the gut microbiota in male BCO1^−/−^BCO2^−/−^ double KO mice. In particular, tomato powder (TP) feeding increased gut microbiota richness and diversity, while it significantly decreased the relative abundance of the genera *Clostridium* and *Mucispirillum*. However, according to the authors, it was not possible to determine the individual beneficial effects that TP’s ingredients (lycopene, apo-lycopenoids, vitamin E, vitamin C, β-carotene, phenolic compounds, and dietary fibers) may exert. A human study focusing on anti-obesity agents showed that lycosome GA lycopene (GAL) or a combination of GAL with dark chocolate (DC) supplementation led to dose-dependent gut microbiota changes, as indicated by an increase in the relative abundance of *Bifidobacterium adolescentis* and *Bifidobacterium longum* [109]. A lycopene-rich diet in postmenopausal women presented a direct and positive correlation with the *Oscillospira* genus, while lycopene consumption was inversely related to the *Pantoea* genus. However, the linkage between lycopene’s contribution to bone and skeletal disorders remains to be ascertained [101]. 

Carotenoids’ potential gut-modulating impact has been also correlated with fatty liver disease. During an animal study, astaxanthin supplementation was able to decrease *Bacteroides* and *Proteobacteria* in six-week-old male C57BL/6J mice, while at the same time elevated the abundance of *Akkermansia*, which is related to potential prebiotic effects against NAFLD [110]. Interestingly, Terasaki et al. [111] demonstrated that an alteration to the fecal microbiota by fucoxanthin was able to prevent colorectal cancer induced by azoxymethane (AOM) and dextran sulfate sodium (DSS) in five-week-old ICR male mice. This intervention exhibited higher concentrations of *Lachnospiraceae* and lower counts of *Bacteroidlales* and *Rikenellaceae*. As a result, fucoxanthin, a marine carotenoid, emerged as a promising therapeutic agent with chemopreventive activity against colorectal cancer [111].

The administration of capsaicin, the carotenoid of red spicy peppers, in obese eight-week-old female C57BL/6J WT and TRPV1^−/−^ KO mice favored the populations of *Prevotella*, *Akkermansia*, and *Bacteroides* and successively the production of acetate and propionate, and at the same time impeded the increase in *Escherichia* numbers [112]. Another member of the red-colored spices family, capsanthin, conferring anti-atherogenic and anti-obesity effects by decreasing trimethylamine *N*-oxide formation, incited the accumulation of *Bacteroidetes*, *Bifidobacterium* and *Akkermansia* populations and suppressed the *Ruminococcu*s class in animal models [113,114]. Notably, a one-month diet of Duroc pigs, enriched with β-carotene, did not elicit any changes in the diversity and richness of gut microbiota [115]. A two-arm, controlled, and randomized trial, where women in mid-pregnancy were enrolled and consumed a carotenoid-rich diet (carrots, apricots, sweet potatoes, bell peppers, oranges, mangos, tomatoes products, etc.) revealed positive correlations of serum α- and β-carotene with the alpha diversity of microbiota. In parallel, beta diversity was affected principally by the intake of β-carotene. Higher carotenoid intake resulted in higher levels of *Ruminococcaceae*, an enterotype for which the association with different dietary patterns is as of yet unclear [116]. 

However, the lack of a representative number of clinical trials involving humans and the inability to explain the mechanisms involved indicate the need for further research in this field.

## 5. Endogenous Lipids

Despite their known contribution to membrane structure and energy storage, lipids are also signaling molecules. Endogenous bioactive lipids are part of a complex network that modulates a plethora of cellular and molecular processes involved in health and disease, while emphasis is placed on their role during inflammation, including gut-related diseases. Thus, it is currently being investigated whether these types of lipids act as promoters or suppressors of inflammation through their interaction with the gut microbiota. Bioactive lipids are: (a) divided into three main families (i.e., eicosanoids, endocannabinoids, and specialized pre-resolving lipid mediators—SPMs) and (b) generated from ω-6 or ω-3 essential polyunsaturated fatty acids (PUFA) precursors, which are esterified into membrane lipids and act by binding and activating specific G protein-coupled receptors (GPRs) [120].

### 5.1. Eicosanoids

**Figure 4:**
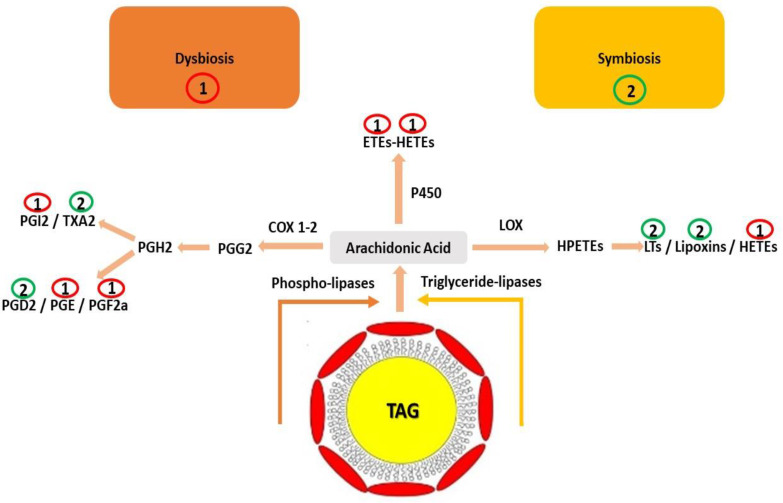
Schematic illustration of different enzymatic pathways (i.e., cyclooxygenase 1,2 (COX-1,2), lipoxygenase (LOX), and cytochrome 450 (CYP450)) of eicosanoids biosynthesis (i.e., prostaglandins (PGE, PGD, PGF, PGI, PGG), thromboxanes (TXs), leukotrienes (LTs), hydroxeicosatetraenoic acids (HETEs), and hydroperoxyeicosatetraenoic acids (HPETEs)) and their role in gut homeostasis [122]. Pink arrows indicate the different biosynthetic pathways, while red and green cycles are associated with gut dysbiosis or symbiosis, respectively.

According to the Lipids Metabolites and Pathways Strategy (LIPID MAPS), eicosanoids are lipid molecules of the fatty acyls group, produced by the oxidation of arachidonic acid. Arachidonic acid (AA) is one of the most important polyunsaturated fatty acids of cell membrane phospholipids, which acts as substrate for a variety of enzymes [121]. These enzymes (i.e., cyclooxygenases (COX), lipoxygenases (LOX), or cytochrome P450), through different biosynthetic pathways, result in the production of different types of eicosanoids (Figure 4) [122,123]. In terms of production, eicosanoids can be formed either by the majority of immune cells or by the intestinal epithelial cell, as the latest findings support [124]. However, even intestinal bacteria may be able to metabolize AA in order to produce eicosanoid metabolites [125]. In particular, LOXs derived from *Proteobacteria* sp. were able to produce various prostaglandins (PGs) through the fermentation of other bacterial metabolites (mostly short-chain fatty acids) in the large intestine [126].

#### Eicosanoids Clinical Effect

A few studies [124,127] revealed that eicosanoids can indirectly affect bacterial populations through their linkage with the normal growth function of the GI tract, as well as their potential role in the regulation of the intestinal epithelial response to injury. However, the exact mechanism, impact, or outcome that each specific eicosanoid has on the gut microbiota may differ significantly (Figure 4). For example, it is believed that PGE2 is related to the appearance of tumors, while PGD2 is characterized by a completely different action [128]. This has a significant impact on intestinal diseases and in particular IBD, since it appears that increased PG production occurs within the mucosa of patients with IBD. Prostaglandins (PG) production indicates a differentiated response, which may lead to a gradual re-shaping of the altered gut microbiota and consequently to a healing effect. Another study demonstrated that the COX-2-PGE2 pathway should be investigated as a target for primary non-responders to tumor necrosis factor (TNF) inhibitor therapy, as well as a prognostic biomarker for TNF inhibitor response in patients with ulcerative colitis [129].

Similarly, mice with leukotriene B4 (BLT4) receptor deficiency appear to be protected in inflammatory disease models of arthritis, asthma, and atherosclerosis. According to Jala et al. [130], when these mice were treated with various tumor factors, the tumor development and mortality were increased, while in germ-free mice, tumors appeared again after fecal transplantation. Microbiota analysis showed a defective host response (e.g., increased *A. muciniphila* sp., *Firmicutes* sp., and decreased *Bacteroides* sp.), reshaping the gut microbiota composition and consequently, promoting tumor growth in the large intestine. Interestingly, it seems that leukotriene inflammatory pathways which are related to tumor growth are clearly dependent on the action of the gut microbiota [130]. Meanwhile, there is evidence showing that LTs can have both positive and negative impacts on bowel-related diseases. For instance, it is widely accepted that the synthesis of leukotriene B4 is enhanced by the colonic mucosa of patients with IBD, helping the development of colitis, while on the other hand, recent data suggest that B4 promotes the intestinal damage repair of epithelial cell proliferation through a low-affinity BLT2 receptor [131].

On the other hand, the most recent approaches correlate LTs and PGs with specific dietary models based on the precursors from which they are derived. For example, the administration of krill oil, which is rich in *n*-3 PUFA such as EPA and DHA, showed pre-resolving properties and the ability to modulate gut microbiota composition (e.g., decreased abundance of *Rickettesiales* sp. and several species of *Lactobacillus* sp.) in a pig microbial-induced dysbiosis model [132]. Another study regarding linoleic acid (omega-6 PUFA) derived from sunflower or safflower showed that either itself or its metabolites (AA, PGE2, and LTB4) were able to enhance IBD [133]. In contrast, soybean or flaxseed a-linolenic acid (omega-3 PUFA) showed that either itself or its metabolic derivatives (EPA, DHA, PGE3, and LTB5) were able to reduce IBD [134]. Overall, the above conflicting results underscore the need for more clinical trials aiming toward the better use (at both the prognostic or therapeutic level) of the axis between nutrition and the role of gut microbiota.

### 5.2. Endocannabinoids

The endocannabinoid (eCB) family presents a complex system of different molecules such as ligands, analogs, and enzymes that are located in many organs and tissues, including the brain and the gut microbiota. According to several studies, eCBs exert immune-regulatory abilities followed by a high specialization that makes them “act on demand”, consequently protecting epithelial barrier integrity and modulating GI motility [122,135]. Among the most studied of these ligands are *N*-arachidonoyethanolamide (AEA) and 2-arachidonoyglycerol (2-AG), which bind and activate type-1 and type-2 cannabinoid receptors (CB1 and CB2), while other eCB members include the following analogs: (1) O-arachidonoylethanolamine (AEA), *N*-oleoylethanolamine (OEA), and *N*-palmitoylethanolamine (PEA). These analogues are synthesized mostly by immune cells using specific enzymes such as the *N*-acylphospathidylethanolamine-hydrolyzing phospholipase D (NAPE-PLD) and diacyglycerol lipase (DAGL).

#### Endocannabinoid System–Intestinal Microbiota Interplay in Gut-Related Diseases

A new scientific field of increasing interest is related to the bidirectional interplay between eCBs and gut microbiota in various inflammatory diseases, such as IBD, rheumatoid arthritis, depression, and consequent pain. However, most of the published studies focus on the differential expression of its components in human IBD. For instance, during a knock-out endocannabinoid degradation study, an improvement in colon inflammation in a colitis C57B1/6 mice model was observed [136]. In contrast, another study, including human and mice stool, identified *N*-acyloethaloamines as a class of metabolites that are elevated in IBD and have the potential to shift the gut microbiota towards a more IBD-like composition (e.g., increased *Proteobacteria* and decreased *Bacteroides*) [137]. Therefore, as Mestre et al. [138] describe, while it is already known that the intestinal microbiome and endocannabinoids interact by affecting the basic functions of each other, there are no corresponding data regarding the mechanisms of action in IBD. Meanwhile, dysregulations of the gut–brain axis, and consequently in eCBs, were related to altered gut microbiota diversity (increased *Lactobacillaceae* and *Erysipelotrichaceae* or decreased butyrate-forming bacteria) in Parkinson’s disease [139]. On the other hand, changes in the gut (reduced microbial alpha diversity) led to the increased excretion of PEA, which in turn led to a more severe clinical condition related to anhedonia/amotivation or other psychological disorders (e.g., depression and schizophrenia) [140]. 

Interestingly, it is believed that the gut microbiota and eCBs can communicate through signals that involve the gut–brain axis for the fine-tuning of energy, lipid, and glucose metabolism [141]. In addition, eCB enzymes also exert a key role in energy homeostasis and metabolism, including metabolic-related disorders such as obesity. In particular, NAPE-PLD regulates fat metabolism and absorption, while its deletion leads to insulin resistance, glucose tolerance, altered lipid and gut microbiota composition (e.g., increased *Alcaligenaceae*, *Bacteroidaceae*, *Clostridiaceae*, *Coriobacteriaceae*, *Erysipelotrichaceae*, and *Lactobacillaceae* families) in an adipose tissue-specific Napepld-deleted mice (cKO mice) model [142]. Notably, OEA and PEA acted as fat sensors through the mediation of the response to high-fat diets, resulting in the control of the thermogenic process as well as the reduction in the increased permeability of the GI tract that often occurs during obesity-driven dysbiosis [141]. 

Although these bioactive lipids appear to provide potential therapeutic abilities, their linkage with gut microbiota composition is indirect. For example, during a 2-day Mediterranean diet in Canadian men and women, specific gut bacterial families (e.g., *Veillonellaceae*, *Peptostreptococcaceae*, and *Akkemansiaceae*) were associated with variations in most *N*-acyl-ethanolamines or 2-AG, independently of fat mass or dietary fatty acid intake [143]. The most commonly accepted mechanism by which the gut microbiota affects the endocannabinoid system involves the regulation of CB2 receptor gene expression. Thus, since the microbiome can affect several gut-related functions through eCBs, the alteration of gut microbiota composition may play a key role in gut-related diseases. This is the main reason why the beneficial role of probiotics on eCBs is being studied. Indeed, during an eCBs-targeted intervention, *L. acidophilus* induced CB2 expression, while the administration of A. muciniphila increased 2-AG [144]. In general, it has been found that *Lactobacillus acidophilus* and *Akkermansia muciniphila* increased the eCBs levels, while *Clostridium* spp was negatively correlated with 2-AG, 2-OG, and 2-PG [145]. However, in order to draw reliable data more human clinical trials are required.

### 5.3. Specialized Pro-Resolving Lipid Mediators: Ideal Molecules for Treating Gut-Related Diseases or Just Another Firework?

A new genus of lipid mediators, also known as specialized pro-resolving lipid mediators (SPMs), are synthesized mostly during inflammation, from ω-6 AA or even further from ω-3 PUFAs, eicosapentaenoic acid (EPA), docosahexaenoic acid (DHA), and docosapentaenoic acid (DPA), and it is believed that they play an important role in a wide range of gut-related metabolic disorders, including diabetes and IBD [146]. The same enzymes (i.e., COX, LOX, and P450) that are involved in eicosanoid synthesis are also associated with the synthesis of these mediators, which takes place after the activation of immune cells such as neutrophils, monocytes, and macrophages. These types of endogenous lipids can: (a) promote the clearance of debris, infective pathogens, and macrophages, which are related to intestinal dysbiosis and (b) inhibit proinflammatory cytokines by enhancing the secretion of anti-inflammatory mediators, resulting in better tissue regeneration, analgesia, and increased functionality [147]. It is also believed that these EPA- and DHA-derived SPMs share similar protective actions with their precursor compounds in modulating innate inflammatory responses, lubricating the GI tract and joints as well as enabling early anticipation and treatment. The most important SPMs are:

- Arachidonic-acid-derived resolvins;
- Eicosapentaenoic-acid-derived resolvins (RvE1-3);
- Docosahexaenoic-acid-derived resolvins (RvD1-6);
- Protectin D1 (PD1);
- Maresins (MaR1 and MaR2);
- Lipoxins (lipoxins A4 and B4, LXA4-LXB4) [148].

**Figure 5:**
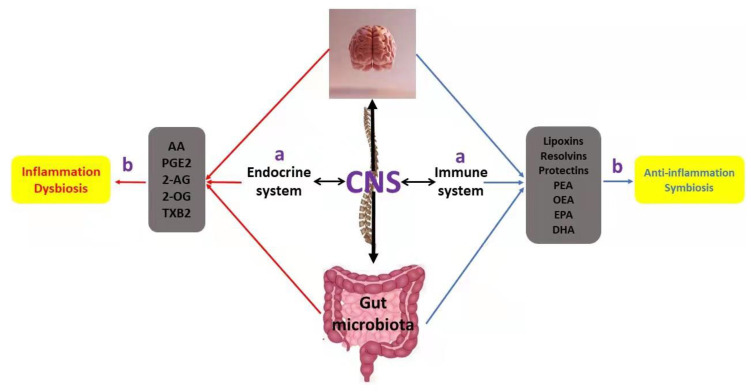
Schematic representation of: (**a**) the complex network also known as the gut–brain axis that involves different types of lipids, the gut microbiota, the central nervous system (CNS), and the endocrine and immune system, as well as (**b**) their impact on inflammation and gut homeostasis. Regarding bioactive lipids, red arrows and the left side indicate their pro-inflammatory activities, including dysbiosis, while blue arrows and the right side demonstrate their anti-inflammatory activities, which in turn drive a sustained net of gut symbiosis.

What differentiates lipid mediators from other signaling-repair lipids is their role as immune-resolvents and not as immune suppressors. This was confirmed by a study in which lipid mediators may have played a pivotal role in the resolution of inflammation and the maintenance of gut integrity [149]. Based on this, it seems that SPMs demonstrate a key role during mucosal infections as well as various gut-related diseases, such as IBD, by promoting the killing of invading pathogens during dysbiosis and enhancing their clearance [150]. As far as inflammatory bowel disease is concerned, the supplementation with ω-3 DPA-derived protectin D1 and ω-3 DPA-derived resolving D5 showed strong protective effects against colitis and intestinal ischemia in eight to ten male C57BL/6 mice [151]. In addition, a recent study using male C57BL/6J mice revealed that MaR1 administration ameliorates the inflammation state in the colonic mucosa and may compensate for changes in the gut microbiota (e.g., increased *P. xylanivorans*) caused by obesity [152]. Similarly, with MaR1, the administration of fish oil or a high dose of resolvin D1 to six-week-old female C57BL/6J obese mice with resulted in the divergence of gut microbiota, which in turn affected body weight [153]. More specifically, microbiota analysis revealed that during resolvin D1 administration *Bacteroides* were increased, and *Desulfovibrio* were decreased, while suppression of the H2S-producing *Deltaproteobacteria* was also observed. Nevertheless, with respect to these promising findings regarding the potential effects of these mediators, clinical evidence with human tested models is still lacking. Overall, endogenous lipids, through signals that mostly involve the gut–brain axis, can play an important role in the design of novel personalized nutrition models by targeting gut microbiota alterations (Figure 5) [154].

## 6. Lipidomics in Current Clinical Interventions: Present Status, Strengths, and Limitations

Although the current knowledge regarding the management of health and disease through the manipulation of the gut microbiome by diet is thriving, the relationship between dietary lipids and microbial populations still warrants more research. In this direction, in order to develop a benchmark for lipidomics, it is necessary to establish a world database in which all the required information (i.e., type of study, samples, techniques, disease, and metabolite outcomes) are recorded. An effort to gather the most recent data regarding lipidomics studies in animal studies and clinical interventions, which include non-polar and endogenous lipids, is presented in Table 3.

It seems that sterols and other metabolites (mainly SCFA) were measured by GC-FID in mostly fecal samples. On the other hand, FSVs and other metabolites were mainly measured by LC-MS in serum or various tissue samples. Continuing, endogenous lipidomics was performed by using LC-ESI/MS or LC-MS/MS in almost every type of biological sample. However, in order to bridge any future gaps in the interpretation and evaluation of the findings of nutrition interventions derived from the implementation of lipidomic techniques, it is substantial to summarize the strong points and the limitations of present clinical studies. The strong points of the lipidomics studies are:

(a) The future design and actualization of cohort studies, which process a vast amount of information, such as lifestyle habits, sociodemographic and anthropometric factors, dietary patterns, and clinical results [164];
(b) The use of holistic –omics techniques (from metagenomics to untargeted metabolomics), the elucidation of novel biomarkers, and the determination of dietary constituents (i.e., carotenoids, vitamins, and sterols) in biological fluids (mainly plasma and feces), which will provide a multifaceted tool in disease diagnosis and treatment [116];
(c) The establishment of evidence in order to create tailored and personalized dietary approaches [155];
(d) The accomplishment of intervention studies, which will include pilot-testing of the dietary patterns that will be then adapted, and will collect more reliable and validated results [99].

On the other hand, the main limitations of current studies are abstracted hereupon:

(a) There is a restricted number of small-sample-size clinical trials concerning human subjects, while valid animal or in vitro models are absent. Therefore, the results of the studies cannot be generalized. In addition, most of the present studies refer to baseline and not to long-term or follow-up interventions (even across the lifespan), which are essential in order to produce representative results [164];
(b) The implementation of non-succinct enrollment criteria and the collection of self-reported questionnaires, related to volunteers’ dietary tracking, may compromise the outcome of the studies due to the past chronic dietary habits or other possible confounders (for instance, Asian populations use plants oils with meat, while European populations consume plant oils in a Mediterranean vegetable-based diet) [99,164];
(c) Inter-individual variations in (socio)genetic factors (i.e., ethnicity or site-specific differences among the same ethnicity) and genetic polymorphisms in non-polar lipids metabolism may imperil the integrity and impartiality of the lipidomics results [116]. For example, populations with less dark skin present a higher risk of vitamin D deficiency [99];
(d) There is a lack of collective knowledge concerning the role of endogenous lipids, especially endocannabinoids and SPMs in clinical studies [154].

## 7. Conclusions

It is apparent that lipids (exogenous or endogenous) have a significant impact on gut microbiota and thus are able to lead the way for potential therapeutic approaches, either by targeting the specific causal pathways of gut-related diseases or by reshaping the composition of beneficial as well as detrimental bacterial populations. Regarding non-polar dietary lipids, phytosterols are considered to be promoters of symbiosis either through the production of SCFAs (i.e., in the case of sitosterols) or through the modulation of gut microbiota composition (i.e., in the case of stigmasterol and campesterol). Meanwhile, sterol metabolites (i.e., coprostanol, methylcoprostanol, and sitostenone) were also found to have a potential impact on gut microbiota composition, while the latest studies highlight their role as potential biomarkers of microbial metabolism. 

In addition, taking into account recent data about the importance of FSVs, it seems that the FSVs–gut microbiota relationship is bidirectional, since FSVs can influence the composition as well as the function of the gut microbiota, while the latter can regulate the status (metabolism, absorption, and functions) of FSVs. This, however, can be a double-edged sword, as it can either promote the necessary symbiosis or induce undesired interactions and enhance the manifestation of pathological conditions (i.e., vitamin K and its association with blood clotting). Thus, in order to elucidate many aspects of this two-way relationship, more human studies, including well-designed dietary or pharmacological approaches, as well as specific bioinformatics tools, are needed. In terms of carotenoid supplementation, numerous clinical interventions have taken place in the last three years. Carotenoids are considered to be phytochemicals with prebiotic-like effects allowing potential therapeutic interventions by regulating the composition of the gut microbiota. However, due to the high diversity of carotenoids, as well as the lack of a representative number of clinical trials involving humans, any kind of generalization would be hasty. 

On the other hand, endogenous lipids are mostly involved in the gut–brain axis, which modulates important biological functions of the host, such as metabolism homeostasis and the immune response. More specifically, the impact of eicosanoids on the gut microbiota is controversial since different types of these endogenous lipids can have completely adverse effects. Notably, these interactions are clearly dependent on the action of the gut microbiota. Furthermore, a new scientific field of increasing interest is related to the effect of the interplay between eCBs and the gut microbiome in various inflammatory diseases, such as IBD and rheumatoid arthritis. Actually, eCBs are able to detect the gut microbiota composition or immune response changes and, consequently, maintain the necessary homeostasis. This also allows the modulation of specific eCB enzymes through microbial interventions (mostly prebiotics) that are associated with positive effects. However, considered to represent the “front line” of endogenous lipids are SPMs, the potential benefits of which are mostly related to the stimulation of inflammation. Surprisingly, these lipids “detect” the increased (pro-inflammatory) cytokines and through the production of anti-inflammatory mediators act as “extinguishers” of inflammation. However, despite these promising results, much more effort is needed, as this evidence has arisen from mostly in vitro or animal studies. 

Regarding the lipidomics status of the presented studies, it seems that each lipid category is investigated using different analytical approaches. In general, GC-FID (in the case of sterols and SCFAs) and LC-MS (in the case of vitamins and carotenoids) are considered to be more suitable for the analysis of dietary lipids. In contrast, endogenous lipid analysis requires an increased resolving and separation power, which in turn will allow a higher sensitivity and broader lipidome coverage. This is why LC-MS/MS, LC-ESI/MS, or, most recently, LC/QC-TOF/MS fit better for the evaluation of endogenous lipids. Despite the analytical limitations in lipidomics so far, the availability of synthetic standards, as well as deuterium-labeled bioactive lipids, now permits the identification and quantification of existing (targeted approaches) or novel metabolites (untargeted approaches) in almost every human biological sample. However, several researchers believe that these studies should be accompanied by an assessment of the composition of the gut microbiome so that specific microbial changes can be associated with the corresponding functions, responses (in case of food intake), or diseases. In any case, lipid analysis must be constantly evolving and able to keep pace with new research data so that it always remains a useful and up-to-date tool for interpreting the complex interactions between the gut microbiota and nutrition. Going back to where we started, it seems that nutrition equally affects the interactions between endogenous lipids and the gut microbiota. However, there is growing evidence that correlates the type of the diet with the precursors (i.e., EPA, DHA, etc.) from which several endogenous lipids are derived. Indeed, more integrated approaches with emphasis on the Mediterranean diet (rich on ω-3 PUFA) can enhance the action of these mediators, while at the same time minimizing any pathological condition that may arise (i.e., cardiological or neurological disorders) according to clinical intervention studies. Overall, despite the differentiation between exogenous and endogenous lipids, the dietary factor remains the most important link that directly or indirectly modulates the intestinal microbiome, allowing prognostic or therapeutic interventions.

## Figures and Tables

**Table 1:** Bacteria phyla and genera in the GI tract.

Major phyla	Stomach	Duodenum	Jejunum	Ileum	Cecum	Colon	Refs.
*Firmicutes*	√	√	√	√		√	[49,50]
*Bacteroides*	√		√	√		√	
*Actinobacteria*	√	√			√	√	
*Fusobacteria*	√				√		
*Proteobacteria*	√			√	√		
**Bacterial genera**	**Stomach**	**Duodenum**	**Jejunum**	**Ileum**	**Cecum**	**Colon**	**Refs.**
*Lactobacillus*			√		√		[51,52]
*Enterococcus*			√	√			
*Streptococcus*			√	√			
*Bacteroides*				√			
*Bifidobacterium*						√	
*Actinomycinae*				√			
*Peptostreptococcus*	√						
*Prevotella*	√						
*Veillonella*	√						
*Rothia*	√						
*Haemophilus*	√						
**Mucosa genera**	**Stomach**	**Duodenum**	**Jejunum**	**Ileum**	**Cecum**	**Colon**	**Refs.**
*Lactobacillus*						√	[53]
*Akkermansia*						√	
*Clostridium*				√		√	
*Enterobacteriaceae*		√	√		√	√	

**Table 2:** Recent studies highlighting carotenoid and gut microbiota interplay through their potential impact in various intestinal diseases and metabolic disorders.

Experimental Model/Disease	Supplementation	Methodology	Carotenoid Impact	Ref.
**Lycopene**				
Male BCO1 ^1 −/−^ BCO2 ^2 −/−^ double KO ^3^ mice/liver cancer	24-week treatment 1st group: HFD + DEN 2nd group: HFD + DEN + Tomato Powder	(1) Liver analysis (2) Lycopene analysis (3) Gut-microbiome analysis	(1) Increased diversity and richness of gut microbiome	[108]
Volunteers, (*n* = 30, 15 women and 15 men)/obesity	1-month treatment 1st group: 10 g DC + GAL 2nd group: 7 mg GAL-MSFA 3rd group: 30 mg GAL-MSFA 4th group: 30 mg GAL-PUFA 5th group: 10 g DC (control)	(1) Biochemical analysis (BMI, pulse rate, and blood diastolic pressure) (2) Gut microbiome analysis	(1) GA lycopene (GAL) had blood-lipid-lowering effects (2) GAL or DC-GAL increase the relative abundance of beneficial bifidobacteria and lactobacilli	[109]
Postmenopausal women (*n* = 92)/bone mineral density	Diet evaluation by a 116-item semi-quantitative food frequency questionnaire	(1) Sequencing of 16S rRNA (2) Fecal samples metabolomics analysis	(1) Increase in *Oscillospira* genus (2) Decrease in *Pantoea* genus	[101]
**Astaxanthin (AST)**				
Male C57BL/6J mice/alcoholic fatty liver disease	12-week treatment 1st group: Normal diet 2nd group: HFD (Control) 3rd group: HFD-Ethanol 4th group: HFD-AST 5th group: HFD-Ethanol-AST	(1) Serum liver analysis (2) Gut microbiome analysis	(1) Decreased Bacteroides-Proteobacteria (2) Increased *Akkermansia muciniphila* which acts as a potential prebiotic during NAFLD	[110]
Male (M)–Female (F) KO and wild-type mice/obesity and diabetes	8-week treatment 1st group: Control diet 2nd group: AST (control + 0.04% AST)	(1) AST fecal analysis (2) Energy expenditure (3) Gut microbiome profile	(1) ASTA affects gut microbiota composition in both (M)-(F) mice (2) The abundance of *Akkermansia* was 385% greater (3) Improvement of metabolic homeostasis only occurs in (M) mice	[117]
**Fucoxanthin (Fx)**				
ICR mice supplied with carcinogenesis agents/colorectal cancer	14-week treatment (3 times per week) 1st group: Oil diet (control) 2nd group: Oil diet + 5% Fx	(1) Gut microbiome analysis (2) Colorectal mucosa analysis	(1) Alteration of gut microbiome by Fx (2) Chemopreventive effect in colorectal cancer	[111]
Male BALB mice/obesity	4-week treatment 1st group: Normal chow diet (control) 2nd group: Normal chow diet + Fx 3rd group: HFD 4th group: HFD + Fx	(1) Cecal and fecal microbiome analysis	(1) Fx changed both cecal and fecal composition (2) Reduced F/B ratio	[118]
		**Capsacinoids (CAP)**		
C57BL/6J (TRPV1+/+) and B6.129X1-Trpv1tm1Jul/J (TRPV1^−/−^) mice/obesity	12-week treatment 1st group: Standard lipid diet (control) 2nd group: HFD 3rd group: CAP + HFD-fed diet	(1) Triglyceride, cholesterol, and insulin analysis (2) Glucose tolerance tests (3) Gut microbiota analysis of feces by 16S rRNA gene sequencing (4) Fecal SCFAs determination by GC-MS	(1) Lower food intake and weight gain, glucose, triglyceride, insulin, and cholesterollevels in CAP + HFD-fed mice (2) Increase in *Akkermansia*, *Prevotella*, *Bacteroides*, *Odoribacter*, *Allobaculum*, and *Coprococcus* in CAP + HFD-fed mice (3) Decrease in *Desulfovibrio*, *Escherichia*, *Helicobacter*, and *Sutterella* in CAP + HFD-fed mice (4) Increase in acetate and propionate in CAP + HFD-fed mice	[112]
C57BL/6J mice/obesity	12-week treatment 1st group: Standard lipid diet (blank control group) 2nd group: HFD (experimental control group) 3rd group: HFD + CAP	(1) Glucose tolerance tests (2) Biochemical analysis, TMAO **** levels (3) Gut microbiota analysis in cecal content	(1) Reduced body weight, serum triglycerides, total cholesterol, low-density lipoprotein cholesterol, and TMAO * in CAP + HFD-fed diet (2) Increase in *Bacteroidetes*, *Bifidobacterium*, and *Akkermansia* in CAP + HFD-fed mice (3) Decrease in *Ruminococcus* and in the ratio of *Firmicutes/Bacteroidetes* in CAP + HFD-fed mice	[113]
		**Various Carotenoids**		
Pregnant women (*n* = 27)	Gestational study at three different time points 1st group: 32-week gestation, pre-intervention 2nd group: 36-week gestation, mid-intervention 3rd group: 6 weeks after child is born, post-intervention Diet containing α- and β-carotene (AC and BC), lutein and zeaxantin (ZL), cryptoxanthin (CR), and *trans-*lycopene (TL)	(1) Plasma and fecal analysis (2) 16S rRNA DNA sequencing of fecal bacteria	(1) AC decreased *Akkermansia* and increased *Phascolarctobacterium* (2) BC increased *Ruminococcaceae UCG002* (3) TL decreased *Akkermansia*, *Escherichia Shigella*, *Phascolarctobacterium*, *Ruminococcaceae UCG002*,*Prevotella* and increase *Ruminococcus* (4) CR increased *Phascolarctobacterium* and decreased *Prevotella* (5) ZL increased *Akkermansia*, *Phascolarctobacterium* and decreased *Prevotella*	[116]
Rats	1-week treatment1st group: Normal diet (control group, *n* = 6) 2nd group: β-carotene supplementation (*n* = 6) 3rd group: Dextran sulfate sodium (DSS), ulcerative colitis model (*n* = 6) 4th group: Dextran sulfate sodium and β-carotene (*n* = 6)	(1) Enzyme analysis of inflammatory cytokines (2) Tissue analysis (3) 16S rRNA sequencing of fecal samples	(1) DSS increased *Proteobacteria* and *Bacteroidetes* and decreased *Firmicutes* and *Actinobacteria* (2) β-carotene reversed these changes (increased *Firmicutes* and *Actinobacteria* and decreased *Proteobacteria* and *Bacteroidetes*)	[119]

^1^ β-carotene -15, 15’oxygenase (BCO1), ^2^ β-carotene -9-10’xygenase^, 3^ double knock out (DKO). * *p* < 0.05, **** *p* < 0.0001.

**Table 3:** Up-to-date lipidomic status of exogenous or endogenous lipids.

Experimental Model	Sample	Analytical Technique	Administrated/Studied Dietary Components	Lipid Species/Biomarkers Detected	Related Disorders	Ref.
Animal model (BALB/c nude mice)	Feces	GC-MS	Sitosterols	SCFAs (↑)	Colocteral cancer	[155]
Animal model (sheep)	Rumen fluid	GC-FID	β-Sitosterol	SCFAs	Rumen acidosis	[66]
Animal model (Syrian Golden hamsters)	Feces	GC-FID	Wood-plant sterols	(a) SCFAs (b) Neutral sterols (cholesterol, coprostanol, coprostanone, campesterol, and dihydrocholesterol) (↑) (c) Acidic sterols (deoxycholic acid, cholic acid, chenodeoxycholic acid, and lithocholic acid) (↑)	High-cholesterol diseases	[67]
Animal model (male Sprague Dawley rats)	Feces	UPLC-QTOFMS ^1^, GC-FID	Phytosterol-ester-fortified skimmed milk	(a) Bile acids metabolic products (i.e., 3alpha,12alpha,15beta-trihydroxy5beta-cholan-8(14)-en-24-oic acid, 2beta,3beta-dihydroxy-6-oxo5alpha-cholan-24-oic acid, 3alpha,11alpha-dihydroxy-12-oxo5beta-cholan-24-oic acid, and (23R)-23-Hydroxy-3,7-dioxo-5betacholan-24-oic acid) (↓) (b) Diglycerides (↓) (c) Novaxenicins A (↓) (d) PI(O-16:0/16:1(9Z)), PG (22:4(7Z,10Z,13Z,16Z)/22:6(4Z,7Z,10Z,13Z,16Z,19Z)), 11R-hexadecanoyloxy octadeca9Z,12Z,15Z-trienoic acid (↓) (e) SCFAs (isobutyric acid, valeric acid, and isovaleric acid) (↑)	NAFLD	[68]
Animal model Syrian Golden (hamsters)	Feces	GC-MS, GC-FID	Soybean sterols	Neutral sterols (coprostanol, campersterol, dihydrocholesterol, and cholesterol) (↑) Acid sterols (deoxycholic acid, cholic acid, chenodeoxycholic acid, and lithocholic acid) (↑) SCFAs (acetic, propionic, and butyric acid) (↑)	High-fat-diet-associated liver damages	[31]
Human, randomized, double-blind, placebo-controlled parallel trial (adult participants)	Serum	GC-FID	Phytosterol-ester-enriched soymilk powder	Fatty acids, DHA, and EPA	NAFLD	[5]
Human, randomized, placebo-controlled crossover trial (adult participants)	Serum, Plasma	GC-MS/MS	Margarine enriched with plantstanol esters	(a) Sitosterol, campesterol (↓) (b) Sitostanol, campestanol (↑) (c) Lathosterol, desmosterol, and cholestenol (no significant changes) (d) 7b-OH-sitosterol, 7b-OH-campesterol, and oxyphytosterol (↓) (e) 7-keto-campesterol (no significant changes)	-	[69]
Human study (adult participants)	Feces	LC-MS/HRMS	-	Cholesterol, coprostanol, cholestanol, sitosterol, 5β-sitostanol, 5α-sitostanol, campesterol, 5β-campestanol, and 5α-campestanol	-	[70]
Human study (adult allograft participants)	Feces	GC-MS	-	(a) Campestanol, coprostanol, and epi-coprostanol (↓) (b) Cholestenone, cholesterylene, and γ-sitosterol (↑)	Kidney failure/kidney transplant	[156]
Animal model (sheep)	H&E-stained tissue samples	LC-MS	High-energy and medium-energy diet vs. normal diet, vitamin A absorption	(a) Viramin E, retinene, cholic acid, litocholic acid, and tauroursodeoxycholic acid (↓) (b) Retinol, glycocholic acid (↑)	Male infertility	[75]
Animal model (male C57BL/6J mice)	Cecal samples	GC-FID	Vitamin A	(a) SCFAs (acetate, propionate, butyrate, and valerate) (b) Branched short-chain fatty acids (BSCFAs) (isobutyrate and isovalerate)	Obesity	[76]
Animal model (BALB/c nude mice)	Plasma, feces	LC-MS/MS	Vitamin E δ-tocotrienol (δTE) and δTE-13′-carboxychromanol (δTE-13′)	(a) Tocotrienols δΤΕ, γΤΕ in plasma and feces (↑) (b) δ-CEHC, sulfatedδTE-13′ with 2 double bonds, sulfated δTE-11′ in plasma (↑) (c) Unconjugated δTE-13′, δTE-13′ with 2 double bonds, 11′-COOH in feces (↑)	Colitis-associated colon cancer	[82]
Animal model (male C57BL/6J mice)	Spinal cord, jejunum, ileum, colon, and duodenum homogenized samples	LC-MS	Vitamin D	Anandamide (AEA) and 2-arachidonoylglycerol (2-AG)	Chronic pain	[95]
Human study (adult allograft participants)	Feces	GC-MS	-	γ- and δ-Tocopherols (↓)	Kidney failure/kidney transplant	[156]
Animal model (male C57BL/6J mice)	Feces	GC-MS	Capsaicin	SCFAs: (a) Acetate and propionate (↑) (b) Butyrate (no significant changes)	Obesity	[112]
Human study (adult allograft participants)	Feces	GC-MS	-	Squalene(↓)	Kidney failure/kidney transplant	[156]
Animal study (male C57BL/6J mice)	Liver tissues	HPLC-UV	Lycopene	(a) IL1β, IL6, IL12a (↓) (b) *Clostriduim*, *Mucispirillum* (↑)	High-fat-diet-promoted hepatocellular carcinoma	[108]
Human double-blinded study (obese participants)	Serum	HPLC-UV	Lycopene	(a) *Bifidobacterium adolescentis and longum* (↑) (b)	Obesity	[109]
Animal study (male C57BL/6J mice)	Fecesepatic and liver tissues	LC-MS, GC-FID	Astaxanthin	(a) *Akkermansia muciniphila* (↑) (b) Plasma glucagon-like peptide (↑) (c) IL-1β (↓)	Inflammation and metabolic homeostasis	[113]
**Endogenous lipids**						
Animal study (C57BL/6J-129/Sv mice)	Colons and small intestines	LC-MS	Eicosanoids	(a) PGE_2_,PGD_2_, 6-keto PGF1_a_, and PGG2_a_, (↓) (b) TXB_2_,15-HETE (no significant changes) (c) Leukotrienes (ND)	Induced intestinal inflammation and tumorigenesis	[157]
Human study (UC verified patients)	Blood	GC-MS	Prostanoids	(a) PGE_2_ in responders receiving a TNF stimulation (↓) (b) PGF2_a_, TXB_2_ (no significant differences) (c) PGI_2_, PGD_2_ (ND)	Ulcerative colitis	[129]
Animal study (male C57BL/6J obese mice)	White adipose tissue	LC-MS/MS	Lipoxin A4	(a) Lipoxin A4 in mice fed a high-fat diet (↓) (b) RvD1, RvD5 (c) Maresin 1	Obesity-induced adipose inflammation/kidney disease	[158]
In vitro and animal study (white Yorkshire-landrace pigs)	THP1 cells Gut luminal and serum	LC/ESI-MS	Short-, medium-, and long-chain fatty acids	(a) (↑) EPA, DHA, and acetate (b) (↓) SCFA	Intestinal inflammation	[132]
In vitro study	Caco-2 cells	LC-MS/MSGC-MS	Fatty acid ethanolamide, FAEs	(a)(↓) PEA, OEA (b) (↓) AEA	-	[159]
Animal study (C57BL/6J mice)	Plasma and adipose tissues	LC-ESI MS/MS	Phospholipids Ceramides Eicosanoids Cannabinoids	(a) (↓) PEA, OEA, and SEA in cKO mice (b) NEFA (no significant differences) (c) (↑) Triglyceride (d)(↑) Cholesterol		[142]
Animal study (C57BL/6J mice)	Colon tissues	LC-MS/MS	Cannabinoids	(a) (↓) 2-AG and 2-OG, and (↑) PGE_2_ in PF-3845 inhibitor mice (b) (↑) 2-AG,2-OG, PGE_2_ (no significant changes) in induced colitis mice (c) NAEs (no significant differences) in both PF-3845 and induced colitis mice	Experimental colitis	[136]
Human study (adult participants)	Plasma	LC-MS/MS	Dietary fatty acid for the determination of the circulation of endocannabinoidome	(a) 7 metabolites of NAEs were found (b) 6 metabolites of 2-MAGs were found	-	[143]
Animal (mice) and human (healthy adult volunteers)	Blood	LC-MS/MS	Impact of resolvins (RvT) in infections	(a) Eicosanoids (b) SPMs (c) Novel 13-series resolvins (RvT1, RvT2, RvT3, and RvT4)	Bacterial infections	[160]
Human study	Urine	LC-MS/MS	Method validation for urinary ω-3 and ω-6 PUFA metabolites	More than 20 PUFA metabolites were identified and quantified	-	[161]
Animal (male mice and human) studies (healthy adults and IBD patients)	Gastrointestinal tissues/plasma	LC-MS/MS	Impact of lipid mediators on intestinal protection	(a) (↑) LTB_4_, PGE_2_, and TXB_2_ in IBD patients (b) (↑) RvD5_n-3 DPA_ and PD1_n-3 DPA_ in IBD patients	IBD	[151]
Human study (healthy adults)	Human plasma/serum	LC-MS/MS	Identification of SPMs through o-3 supplementation	(a) RvE1, RvD1, LXB_4_, 18-HEPE, and 17-HDHA in plasma (b) RvE1, RvD1, AT-LXA_4_, 18-HEPE, and 17-HDHA in serum	-	[162]
Animal study (male C57BL/6J and male Slc:ICR mice)	Feces	CE-TOFMS	Impact of sCSDS ^2^ on the murine intestinal ecosystem	(a) 79 fecal metabolites were identified (b) 16 metabolites were significantly different in sCSDS mice	sCSDS	[163]

^1^ quadrapole time-of-flight mass spectrometry (QTOFMS), ^2^ subchronic and mild social defeat strees (sCSDS).

## Data Availability

The data presented in this study are available on request from the corresponding author.
